# Web-Based Consumer Health Education About Back Pain: Findings of Potential Tensions From a Photo-Elicitation and Observational Study

**DOI:** 10.2196/17130

**Published:** 2020-06-01

**Authors:** Jenny Setchell, Merrill Turpin, Nathalia Costa, Paul Hodges

**Affiliations:** 1 School of Health and Rehabilitation Sciences The University of Queensland Brisbane Australia

**Keywords:** low back pain, lumbar, discourse analysis, qualitative methods, public health

## Abstract

**Background:**

Low back pain (LBP) is a leading cause of disability worldwide, with huge social and economic impact. There is extensive extant literature investigating the efficacy of various management approaches ranging from surgery to psychological interventions to exercise. However, this work has focused almost entirely on efficacy in terms of pain reduction, functional improvement, and psychological changes. This focus has meant that unanticipated social or socio-cultural effects of back pain health care have received little attention.

**Objective:**

This study aimed to scrutinize some of the conceptual tensions inherent in contemporary LBP health care approaches and to highlight their material effects.

**Methods:**

We used a qualitative research design adapted from discourse analysis, which was able to consider key discursive *tensions* underpinning a LBP website. Data collection involved observing the interaction between adult participants with LBP and the website in the following two ways: (1) observational interview, where participants were observed interacting with the website for the first time and asked to discuss their responses to it as they moved through the website and (2) photo-elicitation, where for a month after their first use of the website, people took photographs of what was happening in their lives when they thought of the website and discussed them in a follow-up interview. We used a postcritical discourse analysis approach to examine data produced from these methods.

**Results:**

Our postcritical discourse analysis identified key discursive tensions, including between living with *and* reducing LBP, keeping active *and* resting, and patient choice *and* giving guidance.

**Conclusions:**

Our analysis suggests ways for considering less dominant perspectives without having to discard the benefits of dominant ones. Although the focus of LBP discourses has changed (less biomedical and less about cure), they still hold on to some of the problematic dominant paradigmatic concepts such as biomedicine and individualism. The tensions we highlight are likely to be highly useful for teaching and implementing LBP care across multiple health care settings.

## Introduction

### Background

This paper discusses the use of an adapted discourse analysis approach to consider key nuances and tensions in contemporary approaches to the management of low back pain (LBP). We use the term *postcritical* to delimit a poststructuralist move that blurs boundaries between categories [1-3]. In doing so, we move beyond the discursive dichotomies of *dominant* and *silenced* discourses often constructed in discourse analysis [4]. This blurring allows consideration of how even seemingly contradictory discourses might be fruitfully employed in health care toward beneficial outcomes. We highlight how a postcritical analysis can help to tease out nuances, complexity, and tensions between ideas and approaches to health care management, without needing to dismiss any perspective. For the purposes of this paper, we have used one health care example as a case study: an LBP website that is considered exemplary in its use of both contemporary understanding of pain and the evidence base regarding LBP management.

LBP is widely reported to be the leading cause of disability worldwide [5] and is considered a major global public health problem [6]. Research reports the *burden* of LBP to include considerable direct and indirect costs [7], individual impact [8,9], and wider economic costs [10]. Unnecessary assessments and indiscriminate use of ineffective and potentially harmful treatments have led to the misuse of health care resources [11]. As patient education has been found to be effective for prevention [12] and improvement of LBP [13], access to evidence-based information could potentially lessen the impact of this common condition.

LBP education can be delivered in many ways, such as via health professionals and via public health messaging. People with LBP do not always have access to health education through health care professionals (as they may not deliver it [14]). For this and other reasons, individuals often self-manage their symptoms based on information sourced elsewhere [15,16]. Likewise, service users report a desire for information about their LBP even after consultations with service providers [17]. The internet is a popular and important source of health education for people who experience LBP because of its convenience and high accessibility.

Despite the massive potential of the internet to provide tailored and valuable information, LBP websites are often rated as poor [18], including when evaluated against criteria developed from international guidelines [19,20], and do not meet consumer needs [21]. Given this lack of *trustworthy* information about LBP, a consumer-focused LBP web-based resource was created. The website *MyBackPain* was developed as a research translation output by 2 of the authors (JS and PH) in collaboration with a leading international LBP research organization, key industry bodies, individuals with LBP, and clinicians [22]. The development of the website involved an extensive process of research translation of LBP research, including evidence-based information about 80 types of LBP treatments and contemporary understanding of musculoskeletal pain [22]. The website reflects a postpositivist approach and focuses on the following key messages: (1) enhancement of consumer confidence in self-management and treatment choices, (2) encouragement of engagement in behaviors and attitudes to reduce the burden of symptoms, and (3) reassurance and demedicalization of LBP.

Although the website content is supported by the current LBP literature and its messages reflect an up-to-date understanding of LBP, it is important to consider that MyBackPain may have unintended effects on individuals who experience LBP. For instance, it has been found that emphasis on behavior change, such as engaging in physical activity, can be problematic and might lead to increased shame, guilt, and stigma, which can result in avoidance of healthy behaviors [23,24]. A strong focus on *patient empowerment* can also be problematic and shift the responsibility from society to individuals [25]. Another important issue is that attempts to lessen fear and anxiety can be perceived as devalidating or patronizing by individuals with LBP [26]. It is challenging to present complex information in ways that will benefit the variety of individuals who access sites seeking information about LBP.

### Objectives

The primary aim of this study was to determine how key messages of contemporary LBP health education and their underlying assumptions are taken up by individuals with LBP and consider any potential unintended effects of the messages on the users of the website.

## Methods

### Study Design

We employed a qualitative study design derived from a discourse analysis. We used a combination of 2 data collection techniques: an observational *interview* and photo-elicitation. Data were analyzed using an adapted discourse analysis approach to investigate the (multiple) effects of interactions with the website in the lives of individuals with LBP and how the website’s *key* messages were integrated into consumers’ lives, paying particular attention to any unintended effects of the messages.

### Participant Selection

Participants were recruited through consumer support organizations for people with chronic pain, advertisements in local community centers, contacting participants from previous studies, social media, and word-of-mouth. Inclusion criteria were: (1) self-identification as having (or having had) LBP; (2) English language proficiency; (3) currently living in Australia; (4) aged 18 years and above; and (5) sufficient technological literacy to use a website, learn to use a digital camera, and communicate via conferencing software if required. There were no exclusions based on LBP duration or comorbidities. Efforts were made to ensure inclusion across genders, ages, and representation from both rural and urban participants. We assessed these factors iteratively during data collection: after the first 10 interviews and then again after the next 5 interviews. Recruitment was ceased when a satisfactory level of participant diversity (there were similar numbers of men and women, and there were at least two rural participants, and there were at least two participants in each decade who were aged between 20 and 60 years) and iterative analysis showed few new concepts relating to study aims. The Institutional Medical Research Ethics Committee approved this study.

### Data Collection and Procedure

Participant consent was obtained using a 2-step process. First, all participants were sent the study information via email, and initial written consent was obtained by return email. Second, consent was reconfirmed verbally before the first interview. Data generation consisted of 2 methods:

1. *Observational interview*: For approximately 1 hour, a researcher observed each participant as they interacted with the website for the first time. The researcher asked probing questions during the observation to encourage participants to share their understanding of, and reactions to, the website (eg, “Can you describe to me what you see in front of you?” or “How does what you are looking at make you feel?”). Leading and topic-defined questions were avoided. Interviews were conducted either in person, if participants lived close to the university, or via conferencing software using screen sharing. For the small number of interviews that utilized conferencing software, we used additional prompting questions (in addition to observing which page of the website the participant was on) to ensure we could gain insights into the participants’ interactions. For example, “Can you describe what you are reading or looking at now?” or “Which part of the page were you looking at when you thought that?”
2. *Photo-elicitation*: During the 1-month period following the observational interview, participants were asked to use a digital camera to document moments in which they recalled messaging on the website. A simple digital camera was offered to all participants if they did not already have one. However, all participants elected to use cameras embedded in their mobile phones for convenience. Reminders were sent via text or email twice weekly to prompt participants to take at least one photograph per day (if relevant). Photographs were shared with the researchers and uploaded to a secure location. This methodology allowed the participants to share representations of their lives and experiences through visual content, making the *invisible visible* [27]. The photographs were discussed in an approximately 45-min long interview. Interview questions were semistructured and designed to discuss each photograph to consider how the interaction with the website related to their lives and left room for additional information about participants’ experiences (eg, “Can you discuss why you chose to take this photo?” or “How did you feel when you took that photo?”).

Both sets of interviews were audio-recorded and subsequently transcribed by a professional transcription service. Field notes were written after each interview. Interviewers were all physiotherapists who were trained in interviewing and observational techniques: 2 were females (NC and JS) and 1 was male and external to the research team. JS has a PhD in qualitative research, and NC and the external researcher are PhD students. All data were anonymized during transcription, and photographs and other electronic data were handled securely according to institutional guidelines.

### Methodology and Theoretical Implications

The project was underpinned by an adapted discourse analysis methodology. Discourse analysis considers the way in which language, text, images, or objects produce (or reproduce) certain *realities* or *truths* in relation to power, social, or political inequities [28]. Commonly, critical questions drive the analysis [28]. Our questions were as follows: (1) What messages or *truths* (discourses) were implicitly or explicitly present in the interactions between people with LBP and the website? and (2) What are the material and social implications of these discourses in the broader context of LBP health care?

We intentionally employed data collection methods that produced data on the *interaction* between the website, people with LBP, and their lives. This relational approach is consistent with the new materialist and affective philosophical turn away from purely textual analysis toward consideration of material as well as social effects and understanding of the interrelationship between technology and humans [1,29]. Observation and photo-elicitation added a visual element to the data, expanding sensory awareness and, therefore, induced feelings and thoughts that increase the reflexive process [30] to produce data that were meaningful in the context of people’s lives [31]. Our analysis did not attempt to quantify *how many* or *to what extent* messages were taken up by participants. Rather, our analysis was designed to examine *how* key messages were taken up by participants, consider their underlying assumptions, and any potential unintended material and discursive effects on people’s lives as a result of their interactions with the website.

Rather than highlighting discrete *dominant* or *silenced* discourses (a common approach to critical discourse analysis) in the participant uptake of the website messaging, we conceptualized the discourses on a continuum and in tension. As discussed in the Data Analysis section below, this conceptualization of *tensions* was not a preexisting approach but was produced during our analysis as a way to make sense of our data. This poststructuralist relational conceptualization of a continuum of discourses and *tensions* among this continuum allowed us to move beyond binaries to consider that dominant and silenced discourses might be able to coexist and interact in helpful ways. The dynamic nature of this approach also provided the possibility of considering emphasizing or deemphasizing particular *competing* discourses. In this way, we were able to trace and examine, and not necessarily try to erase (but possibly rework), paradoxes, complexities, and contradictions that are a frequent and perhaps unavoidable part of living with and managing LBP.

### Data Analyses

Analyses were conducted iteratively and concurrently with data collection to allow investigation of new information as the study proceeded. The research team conducted formal analyses of the interview transcript data using a combination of individual and team analysis techniques as follows. First, each team member (all authors) individually reviewed the incoming transcripts to identify concepts relating to the research aims. The subsequent step included team analyses of emergent data in 3 team meetings to refine concepts into key discourses and defining conceptual patterns where *points of tension* between interacting or potentially competing discourses were evident in these data. We developed the concept of *points of tension* during our analysis (ie, it was not a concept we preimposed on the data). Our analysis did not produce any discourses that did not sit among these tensions. The 2 analytic steps were completed 3 times with iterative summative notes made by JS after each cycle and shared with the other authors. These multiple analytical cycles were used to facilitate the identification of patterns and conceptual congruence. Any discrepancies between researchers are included in the reporting of results. Study rigor was guided by Tracy [32], who outlined 8 key markers of qualitative research quality including worthy topic, rich rigor, sincerity, credibility, resonance, significant contribution, ethics, and meaningful coherence. All relevant markers were addressed. Reporting rigor followed the consolidated criteria for reporting of qualitative research [33] with all 28 relevant criteria addressed.

## Results

### Overview

We recruited 15 participants for this study. Participants’ ages ranged from 25 to 68 years, with a mean of 39.5 years. A total of 7 participants were identified as female and 8 as male. All participants were currently employed, except 1 who was studying at a university. Table 1 gives details of the participants’ demographic information. All were interviewed at least once and most were interviewed twice; however, 2 were not available for the second interview (both because of difficulty scheduling the follow-up interview). We included all data gathered from all participants regardless of whether they completed the second interview.

**Table 1:** Demographic characteristics of the study participants.

Demographics		Values
**Age (years)**		
	Mean (SD)	39 (12)
	Range	25-68
**Gender, n (%)**		
	Female	7 (46)
	Male	8 (54)
**Length of time with low back pain (years)**		
	Mean (SD)	14.2 (14.1)
	Range	0.2-33 years
**Current pain level** **(out of 10)**		
	Mean (SD)	3.1 (2.5)
	Range	0-8

Our analyses identified implicit or explicit discourses in the participants’ engagement with the website and identified key points of interaction and potential tension between these discourses that were pertinent to our research aims of determining how key messages embedded in the website were taken up by participants, and the potential unintended material and social implications for individuals with LBP. Textbox 1 presents an overview of these tension points. We discuss each point of tension below using key quotes from the data to illustrate. Participants were distinguished by pseudonyms. As is common in qualitative research, much of the discussion of our findings is included below within this Results section.

**Analysis identified five key tensions between discourses.**

- Reducing lower back pain…living with low back pain
- Providing information…providing guidance
- Keeping active…rest
- Providing information about harmful treatments…feeling okay about choices
- *Human* elements…biomedicine

### Tension 1: Reducing Lower Back Pain...Living With Lower Back Pain

Our analyses highlighted an interaction between 2 different discourses related to managing LBP. These discourses highlighted different *truths* about how to manage LBP. The first discourse was *reducing LBP*—this *truth* could be framed as imperative to work toward reducing, easing, or curing LBP. The second *truth*, *living with LBP,* seemingly conflicts with the first discourse, as it suggests that the focus could be shifted from trying to reduce LBP toward considering how to coexist or thrive while living with LBP. *Reducing LBP* was a prevalent discourse in the data, whereas *living with LBP* was less common. In this section, we first discuss the presence of these discourses in the data and then consider how they interacted and might interact differently.

The research team discussed that these discourses were evident in various ways in the participants’ discussions of the website in the context of their daily lives. The *reducing* *LBP* discourse was particularly apparent when participants were looking at, or remembering, the treatments page of the website. This page presents a list of treatments with descriptions of how much evidence there is to support their efficacy. For example, Jordan, who had only had LBP for 2 months, described that he was looking at this page “to see whether you can relieve my pain immediately” by looking through different management approaches (medication, mind-body exercise, pain thoughts and beliefs, rest and activity, acupuncture, and muscle energy technique). *Reducing LBP* was also evident in that many participants (regardless of the length of time with LBP) took pictures of different management strategies when thinking of the website: for example, Barbara (chiropractic clinic), John (ibuprofen and massage), Martin (2 types of medication), and Sharon (float tank, yoga, chiropractor, osteopathy, and massage). One month later, several of the participants clearly demonstrated that they had remembered elements of what they had read on this page of the website:

It mentioned that paracetamol is not hugely effective. It said Ibuprofen is probably one of the better ones because it is an anti-inflammatory.

John

These data suggest that this discourse is pervasive regardless of the chronicity of LBP.

The *reducing LBP* discourse also arose in more implicit ways. For example, in the following discussion, Dani, who had lived with LBP for more than 10 years, clearly expressed a decision to focus on reducing LBP when discussing her plan to eschew her holiday in favor of focusing on health care:

So I’m going to be spending all this money on Pilates to fix my back and that’s going to be taken out of the holiday fund. But I just realised this morning [smirks], there’s no point going on holiday if I don’t fix my back, because it’s not going to be enjoyable.

The second discourse, *living with LBP*, was less frequently evident in these data and was most often implicitly discussed. As the name suggests, *living with LBP* is different from the first discourse in that the focus now is not on trying to change the LBP but rather working out how to live with it. This could involve approaches toward acceptance, coexistence, or learning to thrive with the condition. For example, Barbara, who also had long-term back pain (>20 years), said in her second interview that she had recently read an article on the Australian national broadcaster’s website (ABC Radio), which reminded her of the messages on the MyBackPain website. She said that the discussion focused on *moving through the pain* and *not restricting your life around the pain.* Albert (with a shorter 2-year history of LBP) also mentioned the website’s messaging to avoid getting scans (such as x-rays and Magnetic Resonance Imagings). The website explains that scans are often not helpful because serious pathology is rarely the cause of LBP and that scans rarely help with diagnosis. Albert suggested that this messaging might make him “feel a little bit more relaxed” about his back pain and that, as a result, he might “face it differently, with a more positive view.” Interestingly, although this message was clear to Albert, he quite strongly disagreed with it and continued to argue that a diagnosis was needed so that the “correct” treatment approach could be taken. The variability in participant responses adds to other research that suggests that accepting less than perfect health states is certainly something that people do, but that levels of acceptance vary with age (increases with age) and severity (decreases with increasing severity) [34]. Although our study was not designed to compare across participant characteristics, our analysis suggests that chronicity might be another factor. Overall, the *living with LBP* discourse appeared to be more contested (and contestable) than the *reducing LBP* discourse, perhaps both in relation to the website and in discursive understandings of LBP more broadly.

There were other times when the 2 discourses were held in considerable tension in the participants’ lives. Returning to Dani’s discussion of whether or not to spend money on Pilates to “fix her back” or to go on holidays, in response to the information on the website, Dani’s discussion put the 2 discourses into competition—she decided that she could not go on holiday (living with LBP) until she fixed her back (reducing LBP). In participants’ responses to the website, there was little attention to concepts such as acceptance of LBP or other forms of the *living with LBP* discourse. Interestingly, in the second interview, it appears that Dani *had* decided to go on holiday. The discourse underpinning her discussion then changed. For example, Dani had taken a picture of a framed photograph she saw in a shop:

I remember the website said that back pain is just one part of your life. It doesn't have to be your life, and is this whole other life around it. Unfortunately, you tend to fall into the trap of, “Oh, my back hurts. I can't do anything.” So when I saw picture of the cow [in a field], it just reminded me of my holiday, and it made me realize that there's a whole bunch of stuff I can do besides just work on my back to reach my goals...just doing things that I used to enjoy doing and getting back to what used to make me happy.

Here, Dani moves away from a focus on changing her LBP (reducing LBP discourse) and refers to different messages on the website than those that were mentioned by people when discussing reducing LBP. She reframes to say that having back pain is acceptable if it is not the only focus of your life. It is not the pain that needs to be fixed or reduced but rather the focus of her life (living with LBP discourse).

At first glance, these discourses appear to be contradictory—one argues for work to reduce LBP and the other argues against a focus on reduction. We suggest, however, that this is not necessarily the case. In fact, it has been argued that it is this very ability to hold 2 apparently contradictory concepts and approach them in a nonlinear way, which is key to being able to adopt the kind of complex thinking that helps to manage health conditions (particularly persistent ones) [35]. We can see a number of practical ways in which this could happen. For example, a person may dedicate some of their time and energy to reducing their LBP—for example, doing things they think help to reduce it (eg, doing exercises or seeking new treatments), and spend some of their time and energy working on living with their LBP (eg, engaging in activities they enjoy or adapting their home environment to make it more comfortable to live with their LBP). This type of *tinkering* with their self-care has been discussed in other research on people’s self-care practices, for example, research on how people living with type 1 diabetes use multiple approaches to manage the complexities of their condition [36,37]. Rather than being problematically contradictory, having both discourses present in health messaging might thus be beneficial to assist patients in managing potential complexities. Clarifying and speaking to this *tension* in a health information resource, such as the MyBackPain website, could help people make more conscious decisions about these potentially competing *truths* and perhaps reduce some of the internal conflict that balancing these truths might bring to the surface.

### Tension 2: Providing Information...Providing Guidance

Another source of complexity in the data was about whether it is the *expert* (in this case, the health resource website—MyBackPain) or the health *consumer* who has the responsibility/choice to decide which approach to LBP management to follow. The tension here was between providing information (ie, presenting choice) and providing guidance as to how to weigh up choices. The complexity of this issue has been discussed in other studies. For example, Pluut [25] highlighted similar discourses in an analytic review of the literature that attempts to define *patient-centered care*. Key discourse**s** identified in that study were *caring for patients*, where it is primarily the *expert* health professional that decides the course of action, and *empowering patients*, where patients are encouraged to make their own choices and decisions [25]. Both approaches have potential pitfalls: if a health resource is too prescriptive, it is a top-down approach that does not allow space for adaptation, individualization, or contextualization. On the other hand, if everything is left up to consumers to decide, this can be an abdication of responsibility, and at worst, neglectful [37]. Similar to Pluut’s findings, our analysis highlights a tension in how the website was taken up by consumers between prescribing *best* courses of action (discourse=*providing guidance*) and leaving choices up to consumers by presenting various options (discourse=*enabling choice*).

One of the participants, Barbara, explicitly discussed the website as placing responsibility for action and decisions on the individuals with LBP rather than on the health professionals (or the website). She expressed this *enabling choice* discourse as positive:

It seems to be a self-help managing pain rather than just a directory of professionals or therapists or people to see. It’s more about self-care which interests me... It doesn’t give you an impression of being a quick fix or having all the answers.

Similarly, when John first viewed the website he said:

It's very upbeat and sort of: “You can take back your life.” “You don't have to stop doing things.” “You just need to manage your lifestyle a bit better.”

Although some of the participants’ responses are positive, this sense of responsibility endowed on the individual can be problematic. It fits with what Foucault [38] would call a *neoliberal agenda*, where control and responsibility are decentralized from traditional forms of power, and instead, people control themselves (or, as Foucault would say *self-disciplining*). One of the reasons why this form of control can be so successful is that responsibility and guilt are closely linked—if something goes wrong, it is then the fault of the individual.

Being responsible for choice can be a burden for some, perhaps particularly those with lower health literacy—people may not have the time or resources to choose well [37]. In some participants’ reactions to the website, there was a sense of disempowerment evident when they were presented with a lot of options with little guidance as to how to understand what they meant. There does not need to be low health literacy for this to be the case, for example, the sense of disempowerment was clear in Jordan’s interview (Jordan has considerable university-level health training); when he was looking through a list of almost 80 treatments, he said with an overwhelmed tone of voice:

There are so many treatments here!

Many treatment types were described, and the level of evidence for each one was given; however, there was little guidance to highlight what *evidence* means (eg, *not enough evidence* was often misconstrued as *does not work*). Expressing the feeling of disempowerment differently, John, who had a positive response when first viewing the website, discussed a photograph he took of a historic jail for the study. When asked why he took that picture, he said:

Having an issue like lower back pain is a bit like being in prison. Because there’s a lot of things you can’t do. There’s a lot of rules you have to abide by. You know, if you really want to take care of yourself, you have to watch what you eat and all that sort of thing.

As explored in other literature [39,40], the burden of responsibility for self-management can be large.

It is important to note that it is inevitably problematic to dichotomize patient choice and guidance as manifesting separately—even when a health resource presents choices, how they are framed, the detail provided about each, the order in which they are presented, and guides people how to act [37]. For example, participant Thomas said he suspected there was a hierarchy in the order in which the treatment options were presented on the website, with the most supported treatment approaches listed higher up in the list. How options are expressed directs people in one way or another—there is inevitably some sense of valence or directionality. It is perhaps unavoidable that a health resource provides some guidance, choices, and education. However, our research suggests that it is important to include in the design of the health resource an explicit consideration of how to balance these options, provision of clear options for guidance or choice, and making it explicit to consumers what is being done.

### Tension 3: Keeping Active...Rest

Another key message that was apparent in the interview data, and was a critical point of tension, was that it is important to keep active (*keeping active* discourse). However, resting was less discussed (*rest* discourse). Like many other participants, Franco said that the main message he took from the website was:

Try to be active, try to be active, try to be active, try to be active. I think that was the first message that I got. I was trying to find guidance, and that’s the first message. The second is that, it’s related to the first one, is to saying that the pain doesn’t necessarily mean that it is damaging more. If not too painful to try to [keep active]. Even if it seems bad, try to do something about it.

Keeping active was seen as a core message by many of the participants. This message is easily recognizable as it is a common contemporary discourse that repeats throughout Western society and health care [40]. Barbara explicitly mentioned this prevalence in her discussion of the website in the context of something she had heard on the radio (mentioned briefly above):

Yeah and exercise and movement... I was actually just reading an article on the ABC website this evening that was talking exactly about this study you’re doing, talking about how moving through the pain, when you have back pain. It’s very much the topic at the moment.

In her first interview, Barbara had added some nuance to the discussion when she first looked through the website:

Personally, I find bed rest difficult because I'm a person who likes doing things and I don't like to be restricted in that way. I do find laying down, especially on the floor, helps my back a lot.

Here, Barbara expressed that some forms of rest are helpful for her. However, overall, rest was rarely highlighted as important to consider/incorporate.

The tension between how much to keep active, in what ways, to what intensity, and how much to rest was little explored in the website. This is a common issue across health care messaging—ignoring the importance of rest is one of the problems that has been highlighted as an unintended outcome of the current focus on *exercise as medicine* [40], which is reproduced in this website. The lack of attention to rest (no one can be active all the time) seemed to contribute to a sense of a lack of clarity for participants as they tried to incorporate messages to remain active into their lives (J Setchell et al, unpublished data, 2020). When people are unable to achieve what is recommended, guilt and shame can be associated with a perceived pressure to keep active [24,41]. For example, in response to reading the following advice on the website, “Research strongly supports returning to normal activities as soon as possible as one of the best ways to recover from back pain. This trains your body’s protection system to not be so sensitive and let you do the things you want to do, without restriction by muscle spasm or pain,” 25-year-old John gave a big sigh and said:

I feel like this is a catch-22. The best way to stop my lower back pain from changing is to stop activities that might set it off. It depends on what the activities are.

Although most participants seemed to incorporate the *keeping active* discourse into their lives, and few incorporated the *rest* discourse, a small number of participants took on a more balanced perspective between the 2 discourses. For example, in her second interview, Megan discussed a photo that she took of a message from a mindfulness app on her smartphone:

Interviewer: ...it says, “Wanting things in the mind to be different is exhausting. Whereas being at ease is a little more peaceful.” Can you explain to me why you took this photo?

Megan: Yeah. It reminded me that there has to be a yin and a yang. You can't constantly be worried or wanting a difference and you need to rest. You need to rest your mind, and you need to rest your body.

### Tension 4: Providing Information About Harmful Treatments...Feeling Okay About Choices

The analysis highlighted a further tension. The website presented information about management strategies that are likely to be harmful or ineffective. The intention of providing this content was to better inform people about the risks of LBP treatments that have strong evidence that they are potentially harmful and/or ineffective (eg, surgery for back rather than leg pain and long-term use of opioid medications). Although alerting people to the risks (side effects, risk of adverse events, and financial cost) of some treatments has obvious positive benefits in terms of warning people about risky or unnecessary treatments, our analysis also highlighted some unintended potential negative effects. The issue seemed to arise when someone had already tried one of the treatments that were said to be harmful/ineffective. For example, in Tiffany’s second interview, she discussed her use of prolotherapy (an expensive and painful treatment that involves multiple injections into ligaments and other tissues around the spine). In her second interview, Tiffany recalled the website’s negative messaging about prolotherapy’s effectiveness (ie, “High quality research suggests that prolotherapy is not helpful for leg or back pain. It is not recommended as a treatment for back pain.”). She explained how she felt when she was at her doctor’s office waiting to receive another course of prolotherapy:

I was disappointed that the website said that prolotherapy wasn’t helpful, even though it was what I was there to have with my specialist...On the website, some of the references to articles were dated. I’ve had back pain for thirty-three years, I want the latest information.

Here, Tiffany discussed her disappointment and, understandably, felt the need to both justify her use of the controversial treatment and discredit the website’s perspective by critiquing the references used. She also discusses the instability of prevailing discourses around LBP:

Like all the things that I learnt about my back thirty-three years ago, they’re telling me the opposite now. I used to treat my pain by trying to ignore it and use distractions, whereas the latest in psychology says you’ve got to accept it and make it part of you. Thirty years ago when I had a spinal fusion, that was the way it was done. But now we’ve moved on and that would be not the way to treat my pain now.

Although this sense of feeling judged or conflicted about previous or current choices after looking at the website was not often apparent in the data, we considered it important. To our knowledge, there is no literature on this topic; however, it is likely that frustration, lack of trust, guilt, and shame can result if people have made these choices in the past/present or when they do in the future. It raised questions such as can we help people manage understandable responses like Tiffany’s to shifting treatment recommendations over time? Is there a way to further invite people to consider the suggestions given on the website in light of the fact that what is believed to be an effective treatment, including what the evidence supports, changes over time?

It was encouraging that 1 participant read the website differently—showing that in some people’s interpretation, there was a good balance between showing that “some treatments are harmful” and “feeling ok about choices.” Barbara said:

I would say that it doesn't discredit any treatment or approach that you want to use. If a treatment or path isn't effective, it's saying that there's not enough evidence, it doesn't just discredit it. It still leaves an opening for people who have tried things and find that they do work. So if they're comfortable continuing to use a certain treatment that works for them, it's not saying “don't do it, you're being ridiculous” or whatever. So I think that's important. I just saw the way that it guides and validates what people have experienced.

If this type of response had been more frequent among participants, this would have been a successful outcome. Ideally, we believe it is unlikely to be helpful to tell people they are wrong to choose treatments that work for them in a health resource or to make them feel bad for using ineffective or harmful treatments, as shame and guilt have long been recognized to be associated with negative health outcomes [42]. Rather, we suggest it is important to be open to a variety of approaches and possibilities that evidence to support or reject particular approaches to management can change [35]. However, at the same time, we want to be clear about the evidence (or lack of) and potential harms/costs of treatments.

### Tension 5: Human Elements...Biomedicine

The final tension was between presentation of biomedical information as well as more *human* aspects of living with LBP. By *human* we mean the nonbiological or biomechanical dimensions of LBP, such as the psychological, social, interpersonal, cultural, or ethical aspects of living with, and managing, health conditions [43], in this case LBP. As a complex and multifaceted approach to LBP is now widely advocated in research to include more than just biomedical elements [6,44], the website presented both biomedical and human aspects of LBP.

Perhaps because of their different perspectives on the relative relevance of the biomedical and human aspects of LBP, participants seemed to be quite divided on whether the website presented a helpful balance of these perspectives. For example, Tiffany spoke about the focus of management strategies presented on the website:

I think the bias is a medical perspective rather than a health and wellness perspective...From what I’ve explored of your website, it didn’t look like it was very favourable to non-medical treatments.

Similarly, when looking through the list of practioners who work with people with LBP provided by the website, Charlotte noted that there seemed to be more practitioners focused on biomedical/mechanical elements than those who attended the *human* aspects of LBP. She added that the professionals listed tended to take an individualistic approach that lacks attention to the broader social/systemic context in which a person with LBP is situated:

I would be more likely to want to see a social worker or a psychologist who can take a systems view [towards LBP management] because I have to manage a lot of different people and medical appointments and so to have someone you can work closely with who draws those people together and draws me into a case management plan. ...They don't have good communication skills. They are lovely people but they don't have the training in micro core communication skills that social workers have so they don't really understand proper empathy and proper communication that patients often need.

To John, even the design of the website felt medical:

it's designed by someone who also does websites for hospitals. It's very much got that feel in the sort of palate and layout.

On the other hand, a small number of participants thought differently. For example, Barbara said she thought the content of the website was “very expansive” and explained the benefits of this by adding:

Instead of having to go through different avenues, through like the scientific, the clinical, the western medicine aspect of things, versus the alternative route. The website seems to include everything.

Overall, it seemed as though most participants experienced the website as attending to more of the biomedical dimensions of living with LBP. Preferably, a more multifactorial approach would better suit current understandings of how to manage persistent conditions such as LBP that affect many aspects of a person’s life.

## Discussion

This study aimed to examine how key messages in a health resource were taken up by participants, to consider their underlying assumptions, and any consider potential unintended effects on people’s lives as a result of interacting with the resource. Our key finding was that there were numerous points of tension that contributed to how participants with LBP were likely to integrate the website messaging into their lives. Our focus on potential unintended negative consequences of this messaging determined that the key points of tension for participants were between (1) *living with LBP* and *reducing LBP*, (2) *keeping active* and *resting*, (3) *providing information* and *providing guidance,* (4) *providing information about harmful treatments* and helping people *feel okay about choices*, and (5) *human* elements and *biomedical* elements. We have highlighted these tensions not only to evaluate this one resource but also to highlight tensions that are likely to be common across management approaches in the field of LBP. Arguably, many of these tensions exist in some form across many aspects of health care, including those beyond LBP. We also believe that, although we focused on health information in the form of a website, the discursive tensions would also be present across different mediums, including face-to-face health care interactions.

This study investigated tensions between different discourses produced in the interaction between a website and the people who use it. Our assumption was that this interaction with websites is not neutral. That is, people do not conduct a neutral examination of the site; they come in with preexisting ideas and experiences that interact strongly with how they navigate the site (eg, what parts of the site they choose to access, what they give the most attention to, and what information they accept or dismiss) and what they learn from the site. People bring their own knowledge and experiences, which interact with the website information in complex ways. Thus, our findings do not attempt to determine the extent to which the website messages are taken up, as they are as much about the individuals who were our participants (and the broader context they live within) as about the health information resource. We, therefore, suggest that readers consider that this study was conducted in Australia, with most participants experiencing LBP over a long period and all participants being employed at the time of the study (1 participant was a student). This would affect the transferability of results across contexts. We acknowledge that the participants might have recalled the website more frequently, or in a different way, because they were knowingly part of a study that included, for example, receiving twice-weekly reminders to make a photographic note of when they recalled the website. Although we did not attempt to examine the amount/extent of website recall, this study context might have affected the emphasis of the tensions we describe.

We suggest that our results can be most useful if the tensions or interactions are not considered as continuums with a beginning and end but rather as a milieu (a middle) where it is not possible to dismiss either aspects of these tensions but acknowledge them explicitly and mix them, perhaps in an amount that is titratable to the individual. That is, it is possible to have a message that speaks to both the concepts to greater or lesser extents. Furthermore, it is also possible that both points can coexist (ie, not necessarily mutually exclusive or antonymic). For example, perhaps it is possible to include evidence about harmful treatments but at the same time discuss potential limitations to evidence, and that it is understandable that at times people choose treatments with little evidence. Indeed, the way forward might be to *include both messages* at once, where possible, as well as making the tensions between them more explicit. Sharing information with that kind of complexity is often easier in formats that allow for more nuances and that engage a human-centered design approach (eg, collaboratively designed videos, artwork, and personal narratives) [45]: messaging that can convey contradictions, emotional content, and contingencies.

## Abbreviations

LBP: low back pain
